# Long-term exposure to ambient NO_2_ increase oral cancer prevalence in Southern China: a 3-year time-series analysis

**DOI:** 10.3389/fpubh.2025.1484223

**Published:** 2025-03-18

**Authors:** Hongbin Peng, Xiaoxia Wang, Ying Liao, Lichong Lan, Danni Wang, Yaohuan Xiong, Ling Xu, Yinxia Liang, Xia Luo, Yunan Xu, Feiyan Li, Hao Chen, Chuanyi Ning

**Affiliations:** ^1^School of Nursing, Guangxi Medical University, Nanning, China; ^2^College & Hospital of Stomatology, Guangxi Medical University, Nanning, China; ^3^The Second Affiliated Hospital, Guangxi Medical University, Nanning, China; ^4^School of Public Health, Guangxi Medical University, Nanning, China; ^5^Key Laboratory of AIDS Prevention and Treatment, Life Sciences Institute, Guangxi Medical University, Nanning, China

**Keywords:** air pollution, oral cancer, distributed lag non-linear model, NO_2_, prevalence

## Abstract

**Background:**

While the correlation between cancer and air pollutants is well-established, research on the delayed effects of NO_2_ on oral cancer remains limited.

**Methods:**

We collected data on nitrogen dioxide (NO_2_) along with diagnosed cases of oral cancer in Guangxi, China, and analyzed the correlation between exposure to NO_2_ and the prevalence of oral cancer.

**Results:**

The study included 1,841 participants diagnosed with oral malignancies, consisting of 1,179 males (64.0%) and 662 females (36.0%), with a mean age of 55.9 ± 14.0 years. The NO_2_ concentration is 20.2 ± 10.4 μg/m^3^. The highest cumulative effects of NO_2_ exposure were observed at a 3-year cumulative lag, with a relative risk (RR) of 1.115 (95% CI: 1.102–1.128). For males, the most pronounced effect of NO_2_ also occurred at a 3-year lag (RR = 1.110, 95% CI: 1.094–1.127). Similarly, among females, the significant cumulative impact of NO_2_ was found at a 3-year lag (RR = 1.123, 95% CI: 1.101–1.145). For individuals under 60 years of age, the cumulative impact of NO_2_ peaked at the same 3-year lag (RR = 1.102, 95% CI: 1.085–1.120). For individuals aged 60 and above, the highest cumulative impact of NO_2_ was also detected at a 3-year lag (RR = 1.132, 95% CI: 1.112–1.152). For the group with normal BMI, the highest cumulative effect of NO_2_ exposure was also observed at the 3-year lag period (RR = 1.289, 95% CI: 1.217–1.365), consistent with the findings for other groups.

**Conclusion:**

These findings suggest a significant lagged effect of long-term NO_2_ exposure on oral cancer, with varying associations between NO_2_ and oral cancer across different ages and genders.

## 1 Introduction

Oral cancer remains a prevalent malignancy affecting the head and neck region (1–3). The increasing incidence of this disease has resulted in significant socio-economic implications (4–6). According to data from the International Agency for Research on Cancer, oral cancer was responsible for approximately 377,000 newly diagnosed cases and 177,000 deaths globally in 2020 (7). Between 1990 and 2019, China experienced a notable increase in oral cancer incidence, with the age-standardized rate surpassing the global average. In 2022, China is expected to report 31,733 new cases of oral cancer, reflecting a rate 1.26 times higher than that of the United States (8). Therefore, it is crucial to explore the risk factors associated with oral cancer to effectively reduce its burden.

The rapid development of the global economy and the transportation industry, coupled with accelerating industrialization and urbanization, resulted in atmospheric contamination that poses a significant risk to community well-being and increases morbidity rates (9, 10). The primary airborne contaminants that threaten human health include fine and coarse particles (PM_2.5_, PM_10_) as well as harmful gases such as CO, NO_2_, SO_2_, and O_3_ (10). Emerging research indicates that exposure to ambient atmospheric contamination is associated with a heightened risk of adverse health outcomes, particularly malignancies such as pulmonary carcinoma (11). Findings from meta-analyses suggest that indoor contaminants also pose a risk for oral malignancies, indicating that issues related to residential air quality may be linked to an increased likelihood of various tumor types beyond lung cancer (12). Evidence indicates that atmospheric contamination adversely affects the oral cavity and its surrounding structures, resulting in issues such as tooth decay, pulp inflammation, and gum diseases (13–16). This suggests that the mouth reacts to exposure to airborne toxins (17). Air pollutants can either dissolve or deposit within the oral environment, leading to inflammation and oxidative damage, which may compromise the body’s immune response and exacerbate symptoms of oral diseases (18). Furthermore, additional data suggest that exposure to atmospheric contamination during pregnancy is correlated with an increased risk of facial and palatal malformations in children (19, 20). Additionally, exposure to airborne toxins may increase susceptibility to premalignant oral lesions and cancers of the mouth (17, 21, 22).

Research conducted in Taiwan has established a significant correlation between levels of PM_2.5_ exposure and an increased likelihood of developing oral malignancies. The findings indicate that exposure to elevated PM_2.5_ concentrations is associated with a 43% higher risk of oral cancer, even after controlling for potential confounding factors such as tobacco use and habitual betel quid chewing (23). Furthermore, a meta-analysis has identified a strong association between indoor air pollution and malignancies of the oral cavity, nasopharynx, pharynx, and larynx (12). This has led some researchers to suggest that home air quality may independently elevate the risk of oral cancer (24). Nevertheless, evidence remains insufficient to substantiate a significant delayed effect of NO_2_ on the incidence of oral cancer. To address these research gaps, we conducted an investigation into oral cancer cases in Guangxi, a province in China. The primary objective of this study is to evaluate whether exposure to NO_2_ has a cumulative delayed effect on the oral cancer. Applying a time-series model, we examined the delayed temporal relationship between NO_2_ exposure and the prevalence of oral cancer.

## 2 Methods

### 2.1 Data source and study population

In this time series analysis, data were obtained from The Affiliated Stomatology Hospital of Guangxi Medical University, the only Grade A tertiary oral specialty hospital in Guangxi. The dataset includes oral cancer diagnosed cases from January 2016 to December 2020, accounting for 16.98% of the total number of hospitalized patients in the hospital. The analysis focused on patients who were recently diagnosed with oral cancer and hospitalized for the first time for therapeutic intervention. All participants in the study were residents of Guangxi, China. For each case, we extracted data on age, sex, marital status, residential address (at the county level), body mass index, and admission date, as shown in Table 1. The study protocol received approval from the Ethics Committee of Guangxi Medical University (No. 2023KY0271). Given that this study involved a retrospective analysis of oral cancer cases retrieved from the hospital’s case management system, informed consent was waived for individual patients.

**Table 1:** Characteristics for the participants of oral cancer.

Variables	Total study population (*n* = 1841)
Age (year), mean, SD	55.9 (14.0)
Sex, *n* (%)	
Male	1,179 (64.0)
Female	662 (36.0)
Marital status, *n* (%)	
Unmarried	104 (5.6)
Married	1,690 (91.8)
Widowhood	26 (1.4)
Divorcee	15 (0.8)
Unknown	6 (0.3)
Occupation, *n* (%)	
Workers	56 (3.0)
Farmer	909 (49.4)
Retiree	252 (13.7)
Jobless	193 (10.5)
Others	431 (23.4)
Oral hygiene status, *n* (%)	
Good	411 (22.3)
Ordinary	585 (31.8)
Bad	805 (43.7)
Unknown	40 (2.2)
BMI (kg/m^2^), *n* (%)	
<18.5	295 (16.0)
18.5–24	1,071 (58.2)
24–28	390 (21.2)
≥28	75 (4.1)
Unknown	10 (0.5)

### 2.2 Air quality data

Data on NO_2_ was derived from the Chinese Academy of Sciences Resource and Environmental Science and Data Center, covering the period from November 1, 2013, to December 31, 2020. Additionally, meteorological data, primarily comprising daily average temperature (T, °C) and relative humidity (RH, %), were obtained from the National Meteorological Science Center,^1^ spanning from November 1, 2013, to December 31, 2020.

### 2.3 Statistical analysis

The data on oral cancer followed a quasipoisson distribution. To evaluate the associations between air pollutants and oral cancer cases, a Quasi-Poisson regression model, based on a generalized linear model and combined with a distributed lag non-linear model (DLNM), was employed. The model utilized daily average concentrations of NO_2_, as predictor variables. When constructing the regression model between daily average NO_2_ concentrations and daily case counts, average temperature was included as a cross-basis function to account for potential meteorological influences. Average relative humidity and indicator variables for day of the week were also incorporated into the model as a covariate. Based on previous studies, a natural cubic spline function with three degrees of freedom (df = 3) was applied to fit the lag-response relationship (25, 26).

Additionally, to ensure the robustness of the model fit, a sensitivity analysis was performed using a dual-pollutant model and by adjusting the degrees of freedom for meteorological variables. This study aims to evaluate the impact of NO_2_ on the prevalence of oral cancer. The findings will quantify the effect of a 1 μg/m^3^ increase in concentrations of NO_2_ in μg/m^3^ on the prevalence of oral cancer within the study population. Relative risk (RR) will be used as the primary metric for this assessment.

Statistical significance was assessed using a two-tailed *p*-value threshold of <0.05. All analyses were conducted using R software version 4.3.1.

## 3 Results

**Figure 1:**
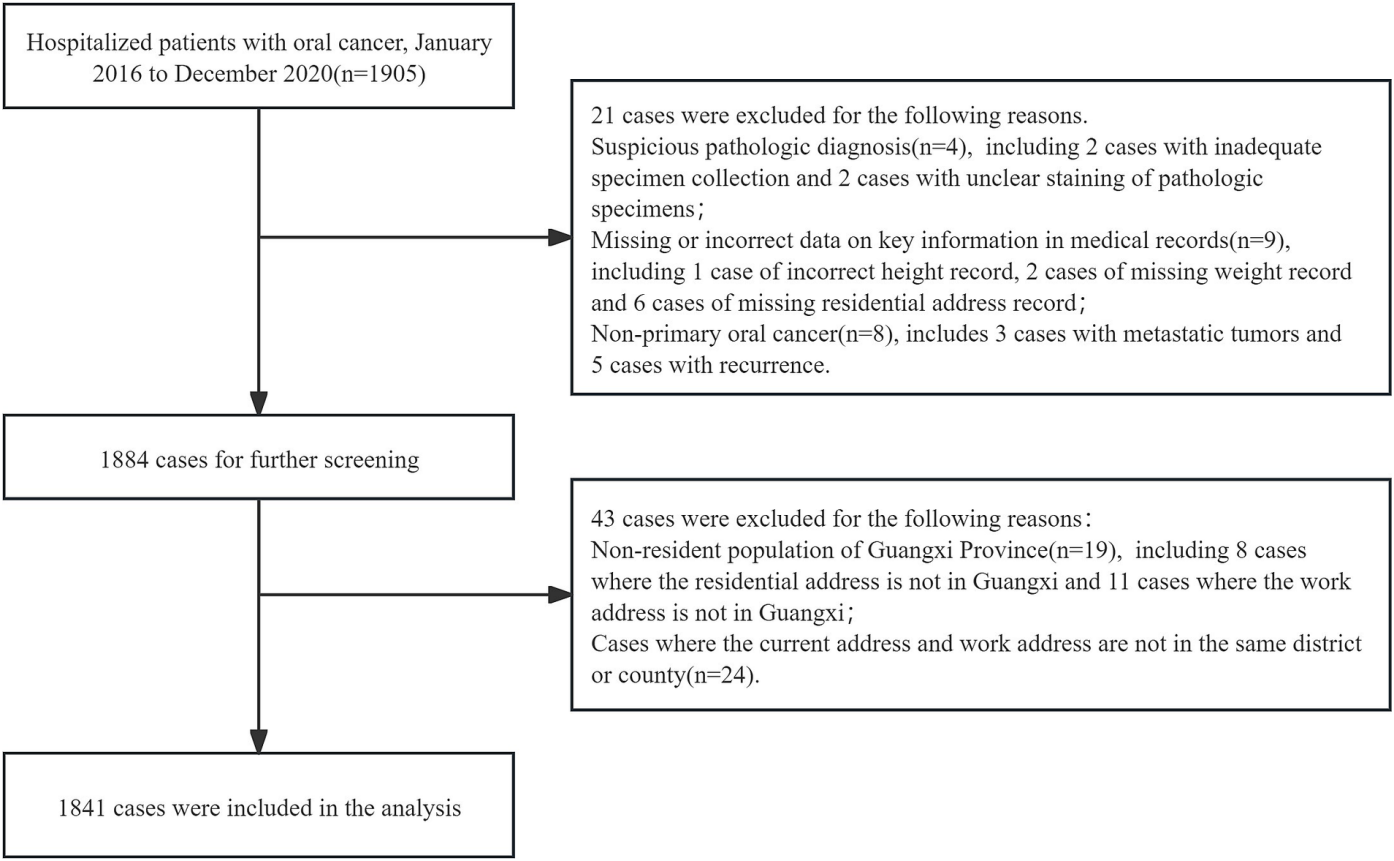
Flowchart of study participants.

**Figure 2:**
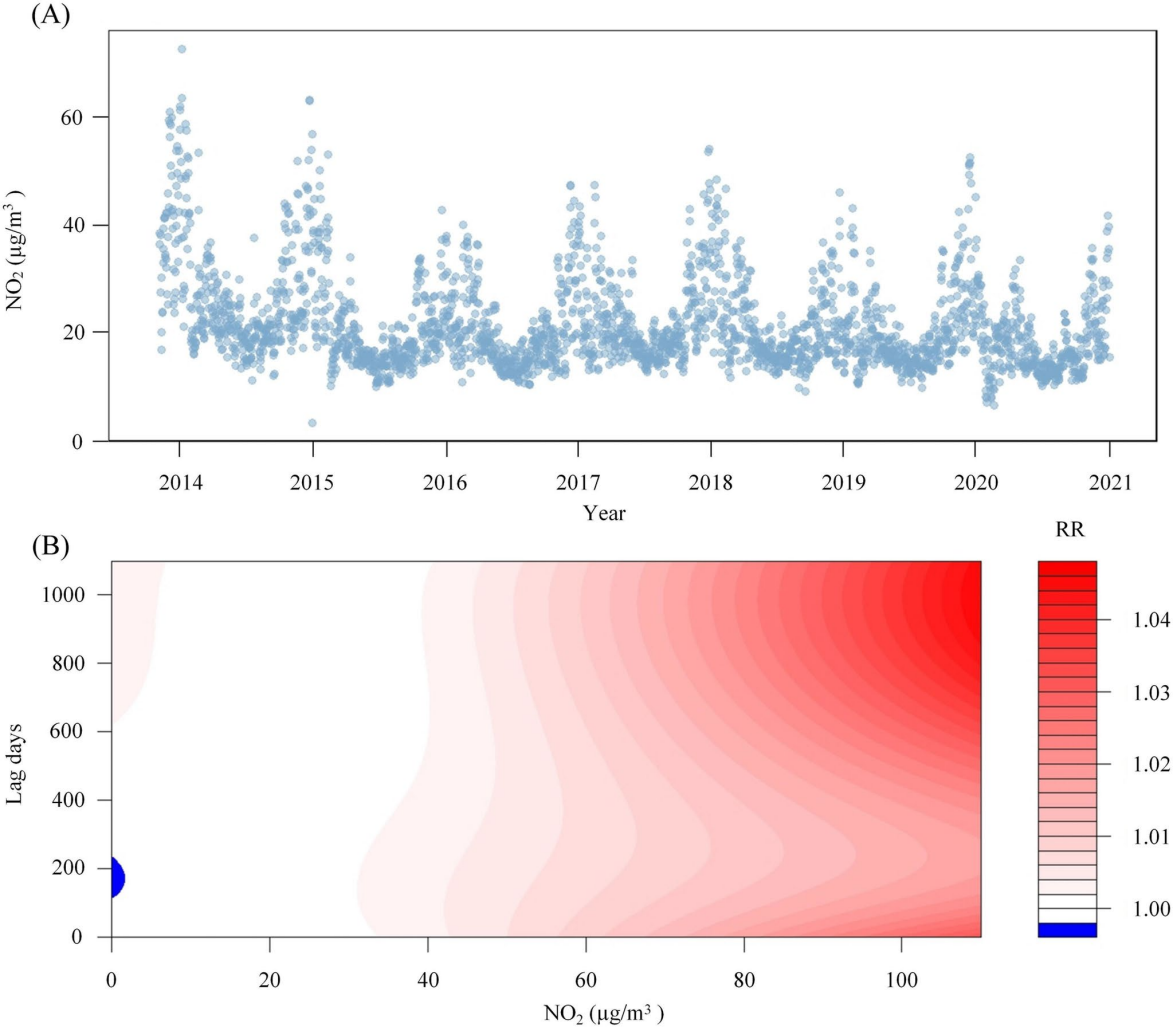
NO_2_ concentration time sequence diagram and the predicted heat map of its impact on oral cancer. Panel **(A)** shows the changes in NO_2_ concentration from 2013 to 2020. Panel **(B)** shows the predicted heat map of the impact of NO_2_ on oral cancer under different exposure times and different concentrations.

**Figure 3:**
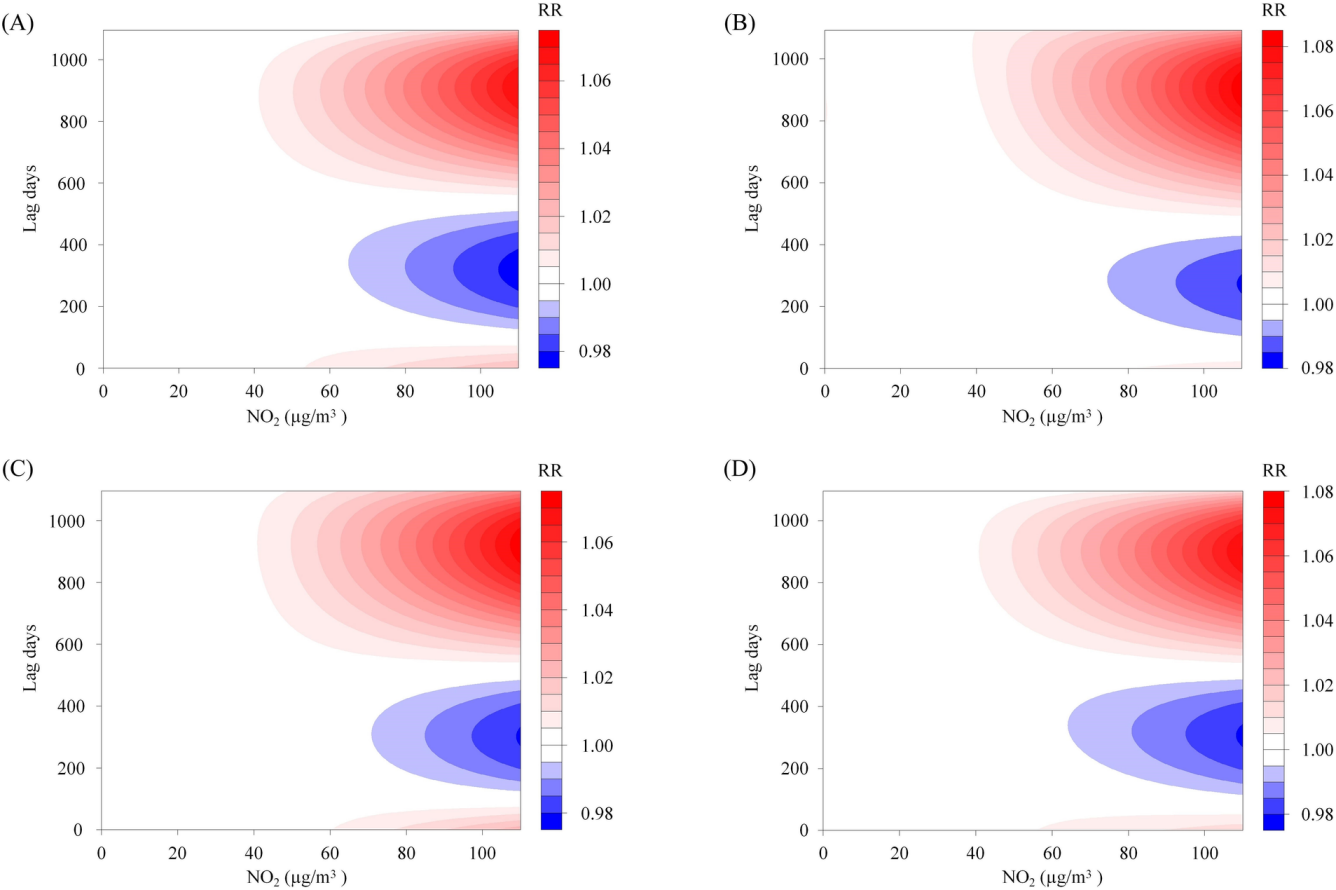
The subgroup analysis of the predictive heatmap of NO_2_’s impact on oral cancer. Panels **(A–D)** represent the predictive heat maps of the impact of NO_2_ on oral cancer in males, females, individuals aged 60 years and older, and those younger than 60, respectively.

This study enrolled 1,841 participants, consisting of 1,179 males (64.0%) and 662 females (36.0%), with a mean age of 55.9 ± 14.0 years. The majority of participants were married (91.8%), and 58.2% had a BMI within the Chinese standard normal range (18.5–24). Furthermore, the distribution of oral hygiene status indicated that 43.7% had bad oral hygiene, 31.8% had ordinary oral hygiene, and 22.3% had good oral hygiene, as show in Figure 1 and Table 1. The NO_2_ concentration is 20.2 ± 10.4 μg/m^3^. Figure 2A illustrates that from 2013 to 2020, the overall trend of NO_2_ concentration exhibited fluctuations, accompanied by seasonal variations. Figure 2B predicts the impact of NO_2_ exposure on the prevalence of oral cancer, indicating an increasing trend correlated with longer exposure durations and higher concentrations. Figure 3 illustrates the effects of NO_2_ exposure and its lagged impacts on oral cancer across various subgroups. The findings indicate that the heterogeneity observed between different genders and age groups is minimal.

Table 2 summarize the associations for patients associated with NO_2_ exposure during different cumulative lag periods and the prevalence of oral cancer. In cumulative lag1-lag3 years, the highest association of NO_2_ with oral cancer was observed at the cumulative lag of 3 years (RR = 1.115, 95% CI: 1.102–1.128).

**Table 2:** Associations between NO_2_ and the prevalence of oral cancer.

Lag years	*β*	SE	RR	95%CI
Lag1	0.070^**^	0.004	1.072	(1.063,1.082)
Lag2	0.100^**^	0.005	1.105	(1.095,1.116)
Lag3	0.109^**^	0.006	1.115	(1.102,1.128)

^**^p < 0.05.

**Figure 4:**
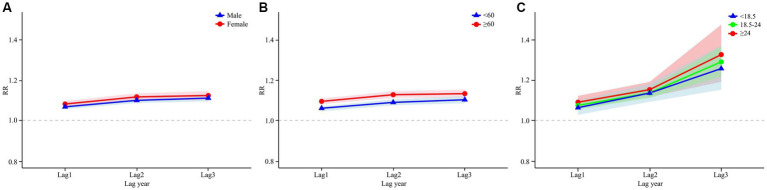
DLNM analysis results stratified by age, gender and BMI. Panel **(A)** shows the results of the gender-stratified analysis, capturing the lag effects of NO₂ exposure on different gender groups over time. Panel **(B)** shows the results of the age-stratified analysis, illustrating the lag effects of NO_2_ exposure on various age groups. Panel **(C)** shows the results of the analysis stratified by BMI, illustrating the lag effects of NO_2_ exposure on different BMI categories.

Figure 4A presents the cumulative lag model illustrating the impact of air pollutants on the prevalence of oral cancer by gender. For females, the maximum cumulative lagged effects of NO_2_ was observed at a cumulative lag of 3 years. Specifically, for each 1 μg/m^3^ increase in the concentrations of NO_2_, the prevalence of oral cancer increased by 12.3% (RR = 1.123, 95% CI: 1.101–1.145). For males, the highest cumulative lagged effects of NO_2_ was seen at a lag of 3 years. A 1 μg/m^3^ increase in NO_2_ concentrations resulted in a 11.0% (RR = 1.110, 95% CI: 1.094–1.127) increase in the prevalence of oral cancer, respectively.

**Figure 5:**
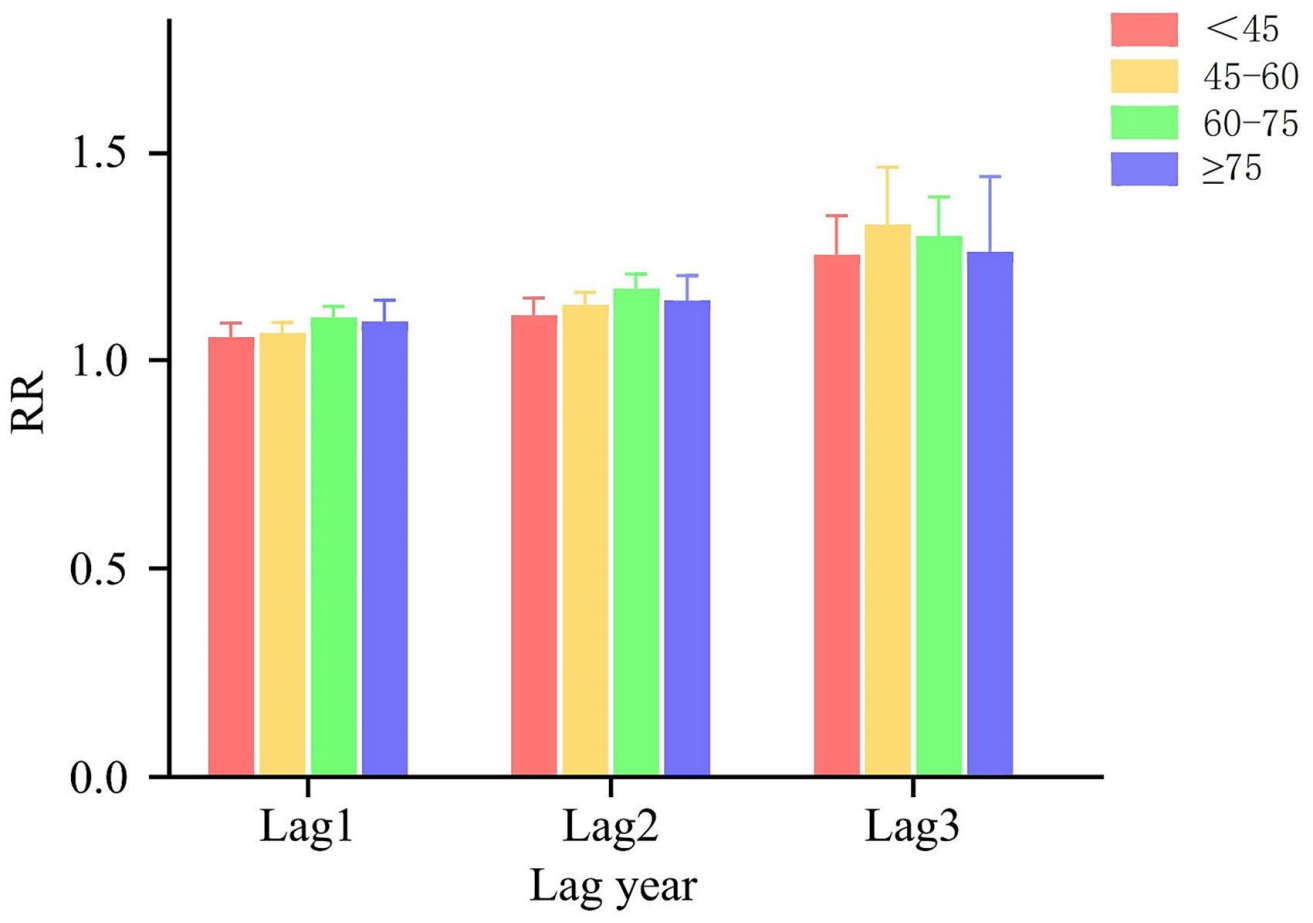
Age subgroup analysis results of DLNM. The results of the DLNM analysis are detailed for these four age subgroups: under 45 years, 45–60 years, 60–75 years, and 75 years or older.

In the age-stratified cumulative lag model for NO_2_ and oral cancer, as illustrated in Figure 4B, the maximum cumulative lag effect of NO_2_ on the prevalence of oral cancer in individuals under 60 years occurs at a lag of 3 years. For each 1 μg/m^3^ increase in NO_2_ concentrations at this cumulative lag, the prevalence of oral cancer increases by 10.2% (RR = 1.102, 95% CI: 1.085–1.120). In the aged 60 and above population the maximum cumulative lag effect for NO_2_ also occurs at 3 years, with an associated increase in the prevalence of oral cancer by 13.2% (RR = 1.132, 95% CI: 1.112–1.152). Figure 5 categorizes all study participants into four distinct age groups: under 45 years, 45–60 years, 60–75 years, and 75 years or older. Across all four groups, the maximum cumulative lag effect of NO_2_ on oral cancer is observed at a 3-year lag, with minimal heterogeneity in the results among the groups.

Figure 4C illustrates the influence of NO_2_ exposure on oral cancer incidence across various BMI categories. The most significant cumulative lag effect of NO_2_ on the three BMI groups—underweight (<18.5), normal weight (18.5–24), and overweight/obese (≥24)—is observed at a cumulative lag of 3 years, with RR of 1.256 (95% CI: 1.152–1.370), 1.289 (95% CI: 1.217–1.365), and 1.325 (95% CI: 1.191–1.473), respectively.

As indicated in Table 3, NO_2_ was selected for the dual-pollutant models at lags of 3 years, respectively. This selection implies that each additional pollutant was introduced at the lag point where the maximum effect was observed in the single-pollutant models. The inclusion of other pollutants did not result in significant alterations to the model outcomes for NO_2_. After integrating SO_2_, CO, O_3_, PM_2_._5_, and PM_10_ into the model, the impact of NO_2_ on oral cancer was quantified as follows: 11.6% (RR = 1.116, 95% CI: 1.105–1.128), 13.4% (RR = 1.134, 95% CI: 1.121–1.148), 12.0% (RR = 1.120, 95% CI: 1.108–1.132), 13.0% (RR = 1.130, 95% CI: 1.118–1.142), and 14.2% (RR = 1.142, 95% CI: 1.128–1.155).

**Table 3:** Results of two pollutants sensitivity analysis.

Air pollutants		RR	95%CI
NO_2_(lag3)		1.115	(1.102,1.128)
	+ SO_2_	1.116	(1.105,1.128)
	+ CO	1.134	(1.121,1.148)
	+ O_3_	1.120	(1.108,1.132)
	+ PM_2.5_	1.130	(1.118,1.142)
	+ PM_10_	1.142	(1.128,1.155)

Supplementary Table S1 details the variations in maximum effect values associated with exposure to NO_2_, analyzed across different degrees of freedom for meteorological factors (df = 1, 2, 4, 5). The findings suggest that altering the degrees of freedom for meteorological factors does not significantly impact the effect values. Consequently, the sensitivity analysis confirms the robustness of the model fit used in this study.

## 4 Discussion

This research explores the potential long-term effects of ambient NO_2_ exposure on the frequency of oral cancer. Previous studies have established a correlation between air contaminants and oral cancer (27); however, our study offers further insights into the impact of chronic exposure to air pollutants on the progression of this disease. Our research findings indicate that the trend in NO₂ concentration changes exhibits seasonal fluctuations. This may be attributed to seasonal variations in wind and precipitation patterns, which can influence the dispersion and deposition of pollutants. Furthermore, our study found that exposure to nitrogen dioxide is associated with an increased likelihood of developing oral cancer, even after controlling for weather conditions. These results enhance our understanding of the relationship between environmental air pollution and oral health. We suggest that long-term exposure to NO_2_ poses risks that extend beyond respiratory health (28), with significant implications for oral well-being. Our findings contribute to the growing body of research highlighting the detrimental effects of air pollution on oral cancer.

Other studies have established a connection between NO_2_ and cancer incidence (29, 30). For instance, a study conducted in Germany found a correlation between NO_2_ exposure and elevated mortality rates, while Canadian research indicated that even low levels of NO_2_ increased the risk of premenopausal breast cancer (31, 32). When NO_2_ is inhaled in excessive amounts, it can cause oxidative damage to the blood, leading to oxidative stress. This process results in the carbonylation of proteins, oxidative injury to cell membranes, and ultimately, apoptosis (33).

Our research indicates a significant correlation between exposure to NO_2_ and the frequency of oral cancer, particularly after a cumulative exposure duration of 3 years. Specifically, 1 year post-exposure to NO_2_, the risk of is estimated at 7.2%, which escalates to 11.5% after 3 years of exposure, suggesting a trend of increasing risk with extended exposure duration. The heightened prevalence of cancer associated with chronic NO_2_ exposure may be attributed to its strong association with combustion byproducts, including volatile organic compounds and carbon-containing materials, many of which are recognized as carcinogenic (34, 35). These findings corroborate conclusions from other studies, which suggest that the delayed effects of atmospheric pollutants may contribute to an elevated cancer risk (29, 36).

The development of cancer is typically a protracted process influenced by the gradual accumulation of atmospheric contaminants, which can inflict enduring damage to human health (37, 38). This cumulative damage progressively heightens the risk of cancer over time. Our research demonstrates that NO_2_ have significant delayed effects on the likelihood of developing oral cancer. Furthermore, as both the duration of lag time and the concentration of pollutants increase, the associated risk of disease escalates correspondingly. Studies indicate that in populations with metabolic abnormalities, the lagged effects of air pollutants can result in chronic vascular damage (39). Such persistent vascular injury may also contribute to the development of various other health conditions (40). Nitrogen oxides can be converted into nitrite in the body, which can subsequently be transformed into carcinogenic nitrosamines. Nitrosamines are regarded as one of the primary contributors to elevated cancer risk. The specific mechanism underlying this process involves complex biochemical reactions; particularly in an acidic environment, the combination of nitrite and amine substances leads to the formation of nitrosamines (41). Exposure to nitrogen oxides may lead to persistent inflammatory reactions, thereby promoting cancer development. Studies have demonstrated a correlation between nitrogen oxides and various types of cancer, which is closely linked to the chronic inflammatory response they induce (42). Systematic reviews have established a robust association between chronic exposure to NO_2_ and urological cancer (43), highlighting the persistent potential impact of air pollution on community health, particularly in relation to cancer risk and other chronic illnesses.

In addition, research indicates that exposure to NO_2_ with a lag of 0 to 2 years, 3 to 5 years, and 6 to 10 years is associated with an increased risk of breast cancer (44). This indicates that individuals exposed to these pollutants may not exhibit symptoms of cancer until a considerable period has elapsed (45). The latency effect may contribute to an underestimation of the health risks posed by air pollution, potentially leading to more severe health complications (46). Given the geographical disparities in air pollution management and economic inequalities, we recommend the establishment of localized guidelines for airborne NO_2_ levels. This approach may yield more significant health benefits by ensuring that economically disadvantaged areas can pursue growth opportunities while allowing more developed regions to continue contributing to global efforts to mitigate and control air pollution (47).

This research conducted stratified analyses by gender and age to identify vulnerable populations exposed to various air pollutants, examining the association between atmospheric contamination and health outcomes within these subgroups. The findings indicate that female demonstrate greater sensitivity to the effects of air pollution, particularly NO_2_, compared to male (48). Several biological and environmental factors may contribute these gender disparities. Female sensitivity to air pollutants is partly attributed to hormonal influences, which may render female more susceptible to pollutants like PM_2.5_ and NO_2_ (49). Additionally, fluctuations in hormone levels during the menstrual cycle and pregnancy may further heighten sensitivity, as these changes can affect airway function and immune response, especially during periods of elevated hormones, such as the premenstrual phase or pregnancy (49). Furthermore, differences in occupational exposure, and lifestyle habits between genders contribute to the observed variability in outcomes (50). The current investigation also found that the impact of air pollution on oral cancer is significantly more pronounced among individuals aged 60 years and older. This heightened effect may be linked to the decreased immune resistance commonly seen in the older adult (51). In comparison to younger individuals, middle-aged and older adults typically exhibit diminished physical function and reduced levels of physical activity, which may lead to increased sensitivity to air pollution (52, 53).

Given the observed lagged effects of air pollution, it is imperative to consider their long-term implications for public health, particularly among vulnerable populations. Policy recommendations should encompass both short-term and long-term strategies aimed at reducing exposure. Enhancing air quality monitoring systems and issuing health advisories during periods of elevated pollution are critical, especially in regions with high-risk groups such as the older adult and children. Furthermore, public health education campaigns can effectively raise awareness about the dangers of air pollution. These campaigns should highlight the importance of minimizing outdoor activities during high pollution events, promoting indoor air purification, and advocating for the use of protective measures such as masks during episodes of significant pollution.

This investigation is subject to several limitations. The primary limitation is the method of exposure assessment, which relied on the residential addresses of participants at the county level. This approach fails to capture detailed personal exposure levels, including individual daily activities, specific locations, and the distinction between indoor and outdoor environments. Consequently, this limitation may introduce potential exposure misclassification, as the actual exposure levels of individuals may differ significantly from those estimated based solely on their residential addresses. In addition, since the cases are from a single-center dataset, it may not accurately reflect the true occurrence of the condition, and the sample may lack representativeness. We recommend that future research employ a prospective design to more accurately assess relevant factors, thereby enhancing the reliability and validity of the findings.

## 5 Conclusion

In summary, this study demonstrates that prolonged exposure to NO_2_ is associated with an increased prevalence of oral cancer, highlighting a significant lag effect. Furthermore, the influence of environmental air pollution on oral cancer is notably greater among females and individuals aged over 60. Consequently, it is essential to improve environmental quality to reduce pollution levels and promote of public health advancements.

## Data Availability

The original contributions presented in the study are included in the article/Supplementary material, further inquiries can be directed to the corresponding authors.

## Footnotes

^1^ http://data.cma.cn/en
