# Supplementary material for: Long-term exposure to ambient NO2 increase oral cancer prevalence in Southern China: a 3-year time-series analysis
Source: Front Public Health. 2025 Mar 18;13:1484223. doi: 10.3389/fpubh.2025.1484223 (PMC11958973; doi:10.3389/fpubh.2025.1484223)
Supplement: Supplementary file 1 [file Table_1.DOCX]

| Table S1 Degree of freedom sensitivity analysis. | | | | | |
| --- | --- | --- | --- | --- | --- |
|  | Model 1 (df=3) | Model 2 (df=1) | Model 3 (df=2) | Model 4 (df=4) | Model 5 (df=5) |
| All (Lag3) | 1.115 (1.102,1.128) | 1.114 (1.101,1.128) | 1.114 (1.101,1.128) | 1.115 (1.101,1.128) | 1.115 (1.101,1.128) |
| Male (Lag3) | 1.110 (1.094,1.127) | 1.110 (1.093,1.126) | 1.110 (1.094,1.127) | 1.110 (1.094,1.127) | 1.110 (1.094,1.126) |
| Female (Lag3) | 1.123 (1.101,1.145) | 1.123 (1.101,1.145) | 1.123 (1.101,1.145) | 1.123 (1.101,1.145) | 1.123 (1.101,1.145) |
| <60 age (Lag3) | 1.102 (1.085,1.120) | 1.102 (1.084,1.120) | 1.102 (1.084,1.120) | 1.103 (1.085,1.120) | 1.102 (1.085,1.120) |
| ≥60 age (Lag3) | 1.132 (1.112,1.152) | 1.132 (1.111,1.152) | 1.132 (1.112,1.152) | 1.131 (1.111,1.152) | 1.131 (1.111,1.152) |
| Note:  Model 1: initial model, the degrees of freedom for meteorological factors were 3  Model 2: the degrees of freedom for meteorological factors was 1  Model 3: the degrees of freedom for meteorological factors were 2  Model 4: the degrees of freedom for meteorological factors were 4  Model 5: the degrees of freedom for meteorological factors were 5 | | | | | |
